# Treating Attention-deficit/hyperactivity disorder During Pregnancy and Breastfeeding

**DOI:** 10.1192/j.eurpsy.2023.433

**Published:** 2023-07-19

**Authors:** A. S. Vieira, H. Santos, I. Valada

**Affiliations:** Centro Hospitalar Psiquiátrico de Lisboa, Lisboa, Portugal

## Abstract

**Introduction:**

Attention-deficit/hyperactivity disorder (ADHD) is one of the most common neurodevelopmental disorders, and in the majority of patients persists into adulthood. There is a lack of data regarding the risks of ADHD medication during pregnancy and breastfeeding. While some women may be able to discontinue without adverse effects, others may experience significant functional impairment. Due to the rising number of ADHD medication prescribed to women at child-bearing age, it is important to determine which medications can be considered relatively safe in pregnancy and lactation.

**Objectives:**

We aim to review recent evidence on the risks of stimulant and non-stimulant treatment in pregnancy and lactation.

**Methods:**

Literature review on the topic through PubMed and Google Scholar using the search terms: “ADHD”, “ADD” , “Pregnancy”, “Lactation OR breastfeeding”, “Stimulants”, “Methylphenidate OR Amphetamine OR lisdexamfetamine OR atomoxetine OR modafinil”. Only original research papers written in English were included.

**Results:**

We identified twelve studies investigating the use of ADHD medication in pregnancy and four studies regarding lactation. Most of the studies did not find an elevated risk for congenital malformations by treatment with methylphenidate or medical amphetamines during pregnancy. A report suggested a moderate risk for congenital defects in infants exposed to modafinil in utero. The teratogenic effects of atomoxetine and guanfacine have not been investigated. Regarding lactation, only case reports and case series were found. Methylphenidate seems to be safe, with little transfer into breast milk and no reported adverse effects for the baby. Amphetamines transfer into breast milk and reach relatively high concentrations, and although the overall risk for intoxication seems to be low it cannot be fully excluded.

**Conclusions:**

Prescription of ADHD medication to pregnant and lactating women should be considered after an individual risk-benefit estimation. In severe cases, when medication cannot be discontinued, the overall risk for adverse outcomes seem to be relatively low. More higher quality studies are needed on the topic.

**Disclosure of Interest:**

None Declared

